# Supplementary material for: Spatio-temporal evolution and convergence patterns of E-commerce development in China: Regional disparities and policy implications
Source: PLoS One. 2025 Aug 29;20(8):e0330066. doi: 10.1371/journal.pone.0330066 (PMC12396765; doi:10.1371/journal.pone.0330066)
Supplement: Attachment — Submitted filename: Response to Reviewers.docx [file pone.0330066.s002.docx]

## Response to Reviewers

**Dear Ms. Vanessa Carels,**

We are truly grateful for the reviewers' insightful comments on our manuscript. Our point-by-point responses to their valuable suggestions are provided below.

**Response to Reviewer #1**

1. In response to Reviewer 1's suggestion, we have revised the title accordingly.

2. We have incorporated a more detailed description of the data within the abstract.

**Response to Reviewer #2**

1. We have drawn a clearer and more explicit comparison between the present study and similar prior research.

2. We have discussed the potential biases that may arise from imputing missing data using the interpolation method.

3. All figures within the manuscript have been ensured to possess a resolution of 300 dpi and have undergone verification using PACE.

4. We have meticulously corrected all identified linguistic and stylistic errors throughout the manuscript.

**Response to Reviewer #3**

1. The research motivation for this study, along with the significance of comprehending spatio-temporal evolution characteristics and convergence trends, has been elaborated upon in the second paragraph of the Introduction.

2. Statistical analyses indicated no discernible impact of the pandemic on the spatio-temporal distribution characteristics of e-commerce in China. Therefore, a separate, detailed investigation into the pandemic period was not pursued in this study.

3. The findings of this study offer significant implications for policy-making and the sustainable development of e-commerce.

**Response to Reviewer #4**

1. We have clarified in the manuscript that the spatial weight matrix employed is an adjacency matrix, with a corresponding explanation provided.

2. A detailed rationale for the selection of control variables has been included in the manuscript.

3. We confirm that the datasets utilized in this research have been successfully uploaded.

4. We have elaborated on the characteristics of the LISA cluster map, providing a detailed discussion in conjunction with factors such as infrastructure.

5. The primary limitations of the model have been explicitly outlined and discussed.
